# Peroxiredoxin 3 regulates breast cancer progression via ERK-mediated MMP-1 expression

**DOI:** 10.1186/s12935-024-03248-x

**Published:** 2024-02-06

**Authors:** Pei-Jou Chua, Suet-Hui Ow, Cheng-Teng Ng, Wan-Hong Huang, Jie-Ting Low, Puay Hoon Tan, Michael W.Y. Chan, Boon-Huat Bay

**Affiliations:** 1 Department of Anatomy, Yong Loo Lin School of Medicine, National University of Singapore, Queenstown, 117594 Singapore; 2 Department of Biomedical Sciences, National Chung Cheng University, Min-Hsiung, Chia-Yi, 62102 Taiwan; 3 Epigenomics and Human Diseases Research Center, National Chung Cheng University, Min-Hsiung, Chia-Yi, 62102 Taiwan; 4 Division of Pathology, Singapore General Hospital, Singapore, 169608 Singapore; 5 Center for Innovative Research on Aging Society (CIRAS), National Chung Cheng University, Min-Hsiung, Chia-Yi, 62102 Taiwan; 6 Present Address: Luma Medical Centre, Royal Square, 329565 Singapore

**Keywords:** Breast carcinoma, Peroxiredoxin, MMP-1, ERK signaling

## Abstract

Peroxiredoxin 3 (PRDX3), a mitochondrial hydrogen peroxide scavenger, is known to be upregulated during tumorigenesis and cancer progression. In this study, we provide evidence for the first time that PRDX3 could regulate cellular signaling pathways associated with Matrix Metalloproteinase-1 (MMP-1) expression and activity in breast cancer progression. We show that shRNA-mediated gene silencing of PRDX3 inhibits cell migration and invasion in two triple-negative breast cancer cell lines. Reciprocal experiments show that PRDX3 overexpression promotes invasion and migration of the cancer cells, processes which are important in the metastatic cascade. Notably, this phenomenon may be attributed to the activation of MMP-1, which is observed to be upregulated by PRDX3 in the breast cancer cells. Moreover, immunohistochemical staining of breast cancer tissues revealed a positive correlation between PRDX3 and MMP-1 expression in both epithelial and stromal parts of the tissues. Further pathway reporter array and luciferase assay demonstrated that activation of ERK signaling is responsible for the transcriptional activation of MMP-1 in PRDX3-overexpressed cells. These findings suggest that PRDX3 could mediate cancer spread via ERK-mediated activation of MMP-1. Targeted inhibition of ERK signaling may be able to inhibit tumor metastasis in triple-negative breast cancer.

**Supplementary Information:**

The online version contains supplementary material available at 10.1186/s12935-024-03248-x.

## Introduction

The most prevalent female cancer is breast cancer, which affects 2.26 million women worldwide with an estimated 685,000 women who perished from this disease in 2020 [1]. Tumor recurrence and metastatic spread are the leading causes of high breast cancer mortality [2, 3]. Early detection of breast cancer is critical in determining treatment options and survival outcomes. Classical tumor markers which include Estrogen receptor (ER), Progesterone receptor and Human epidermal growth factor receptor 2, and emerging biomarkers, such as Ki67 protein and multigene signatures, allow for the screening of patients and selection of appropriate therapy [4–6]. However, triple-negative breast cancer (TNBC) still presents a clinical challenge due to its aggressiveness and the lack of targeted therapies [7, 8]. Hence, there is a need to develop novel therapeutic strategies to improve patient outcome in this subgroup of breast cancers.

Peroxiredoxin 3 (PRDX3) belongs to a family of antioxidants, which has multiple isoforms with expression that is spatially and temporally regulated, thereby allowing them to perform a plethora of functions in different sub-cellular compartments [9, 10]. The active thiol group present on the molecule offers protection against oxidative damage from reactive oxygen species (ROS), which is known to play significant roles in carcinogenesis [10, 11]. PRDX3, a scavenger of hydrogen peroxide (H_2_O_2_) in mitochondria, is known to detoxify ROS generated so as to maintain homeostasis in this cellular organelle. High levels of ROS can induce DNA mutations leading to carcinogenesis or trigger the apoptosis pathway leading to cell death [12, 13]. However, at physiological levels, ROS are signal transducers which are involved in signaling and regulation of cellular activities such as cellular proliferation [14]. Cancerous cells with elevated PRDX3 expression would confer an advantage as high PRDX3 could facilitate rapid detoxification of ROS generated, protecting the cells against oxidative damage and apoptosis [15]. PRDX3 has also been documented to be involved in tumor progression in several human cancers [15–20], however its role in breast cancer is not fully explored.

Hence, in this study, we investigated the possible mechanism of PRDX3 in breast cancer progression. PRDX3 overexpression promoted cancer cell migration and invasion, while depletion of PRDX3 reversed this phenomenon in triple negative breast cancer cells in vitro. This phenomenon is attributed to the upregulation of MMP-1 via ERK signaling pathway activation.

## Materials and methods

### Cell culture

Human breast cancer cell lines, MDA-MB-231 (ATCC®HTB-26™) and BT-549 (ATCC® HTB-122™) were obtained from American Type Culture Collection (Rockville, MD, USA). Both cell lines were grown in RPMI-1640 containing 10% fetal bovine serum (FBS), with the addition of 0.023 U/ml insulin for BT-549 cells.

### Silencing of PRDX3 by shRNA or siRNA

MDA-MB-231 cells were transfected with shRNA targeting PRDX3 (shPRDX3) or scrambled shRNA cassette (sh-NT) using Turbofectin 8.0 transfection reagent (Origene Technologies, Inc., Rockville, MD, USA), according to the manufacturer’s instruction. Cells were then selected by puromycin dihydrochloride. siRNA transient transfection was conducted in BT-549 cells using a targeting pool of 4 siRNAs from Dharmacon (Chicago, IL, USA) as described previously [21].

### RNA extraction, reverse transcription, and quantitative real-time RT-PCR

Total RNA was extracted from cells using the RNeasy Mini Kit (Qiagen Gmbh) and 1µg of RNA was reversed transcribed into cDNA using the AffinityScript QPCR cDNA Synthesis Kit (Agilent Technologies) according to manufacturer’s instruction. Quantitative real-time RT-PCR was performed using Applied Biosystems 7900HT Fast Real-Time PCR System (Life Technologies). The primer sequences used were as follows: PRDX3-F: 5’-GCA GAT TTC CCG AGA CTA CG-3’; PRDX3-R: 5’-TAG GAG AAT CCG GTG TCC AG-3’; MMP-1-F: 5’-TCA CAC CTC TGA CAT TCA CCA-3’; MMP-1-R: 5’-AAT GAG CAT CCC CTC CAA TAC-3’; and GAPDH-F: 5’-GAA GGT GAA GGT CGG AGT CAA-3’; GAPDH-R: 5’- TGC CAT GGG TGG AAT CAT ATT GG-3’. The relative expression of genes was derived using the comparative CT method and normalized to the expression level of reference gene GAPDH.

### Migration and invasion assays

Boyden migration inserts or Corning® BioCoat™ Matrigel® Invasion Chamber (Corning Inc., Corning, NY, USA) were used. Cells were harvested in serum free culture medium and seeded into the inserts. The lower wells contained complete culture medium supplemented with 10% FBS. After 24 h, the cells were methanol-fixed before staining with 0.5% (w/v) crystal violet. Cell in the upper chamber that did not migrate were removed by a moist cotton swap. The chambers were subsequently viewed under a microscope and the average number of cells that migrated/invaded were tabulated.

### Western blot

Whole cell lysate (WCL) was extracted using RIPA buffer (Thermo Fisher Scientific) in the presence of Halt™ protease and phosphatase inhibitor cocktail and EDTA (Thermo Fisher Scientific). For subcellular fractionation, it was prepared using NE-PER® Nuclear and Cytoplasmic Extraction Reagents (Thermo Fisher Scientific) according to the manufacturer’s protocol. To extract protein from conditioned medium (CM), culture supernatant was first centrifuged at 1000 rpm for 5 min to remove cell debris. The supernatant was then concentrated with Amicon Ultra-15 10 K Centrifugal Filter Units (Merck Millipore, Burlington, MA) by centrifuging at 5000 g for 45 min at 4^o^C. Protein concentration was determined using a Bio-Rad protein assay kit (Bio-Rad laboratories, Hercules, CA). 30 µg of denatured protein samples were separated by sodium dodecyl sulfate polyacrylamide gel electrophoresis (SDS-PAGE) and transferred to polyvinylidene difluoride (PVDF) membranes. 5% (w/v) bovine serum albumin (BSA; Santa Cruz Biotechnology, Santa Cruz, CA) was used as blocking solution to block unspecific antibody binding sites. The membrane was then incubated with primary antibodies at 4^o^C overnight. The primary antibodies used were as follows: rabbit polyclonal anti-phospho-c-Jun and rabbit polyclonal anti-total c-Jun (1: 1000, Cell Signaling Technology, Danvers, MA), rabbit polyclonal anti-MMP-1 (1: 2000, Proteintech Group, Chicago, IL), mouse monoclonal anti-GAPDH (1: 2000, Santa Cruz Biotechnology) and mouse monoclonal anti-Lamin B1 (1: 2000, Proteintech Group). The blots were then incubated with the corresponding secondary antibodies conjugated to horseradish peroxidase (Sigma-Aldrich) with dilutions as shown in Table S1, at room temperature for 1 h. The immunoreactive bands were visualized with enhanced chemiluminescence (Thermo Scientific) and quantified with a densitometer (Bio-Rad laboratories).

### MMP-1 activity assay

The enzyme activity of MMP-1 was quantitated using the Fluorokine E Human Active MMP-1 kit (R&D System, Inc.) according to manufacturer’s instructions. The culture supernatant were collected and incubated in a 96-well plate coated with monoclonal antibody specific for MMP-1. The activity of MMP-1 in cells were measured by performing a standard curve using an MMP-1 standard and the chemical activator p-aminophenylmercuric acetate (APMA) which activates pro-MMPs. The quenched fluorogenic substrate was added following a wash. The cleavage product by active MMP-1 was determined using a microplate reader with an excitation wavelength of 320 nm and emission wavelength of 405 nm.

### Luciferase reporter activity assay

The Cignal Finder 10-pathways Reporter Arrays kit (Qiagen) was used to detect the activity of ten pathways associated with cytotoxicity and cellular stress activation upon PRDX3 knockdown or overexpression. 8 × 10^4^ cells were reversed transfected to the plate array using Attractene transfection reagent (Qiagen) according to manufacturer’s protocol. After 24 h of transfection, the dual luciferase assay was performed using Dual-Luciferase Reporter Assay system (Promega Corporation) and measured using SpectraMax M5 microplate reader with Firefly luciferase at 560-580nM and Renilla luciferase at 480nM, (Molecular Devices). Experiments were done in quadruplicates and repeated thrice. The relative luciferase unit (RLU) was calculated as the ratio of firefly luciferase to renilla luciferase value. The fold change was determined by dividing the RLU of PRDX3 knockdown/overexpression with the corresponding sh-NT or EV control.

### Human phospho-MAPK proteome profiler array

Relative expression levels of phosphorylated MAPK and other serine/threonine kinases were examined using the Proteome Profiler human phospoho-MAPK antibody array kit (R&D Systems). 100 µg of protein lysate was used for the array according to manufacturer’s protocol. The intensity of each spot representing individual phosphorylated protein was determined using a densitometer.

### Treatment with ERK inhibitor in breast cancer cell line

3 × 10^5^ cells were treated with 500nM or 1µM of SCH772984 (Cayman, Ann Arbor, Michigan) in a 6-well plate for 24 h. Cells were then harvested for RT-qPCR to examine the relative expression of PRDX3 and MMP1.

### Clinical materials from breast cancer patients

Four-µm thick tissue microarrays (TMAs) were constructed from a total of 202 cases of invasive ductal carcinoma (IDC) breast cancer tissues (with 67 cases of adjacent normal breast tissues) collected from patients who were diagnosed with IDC at the Singapore General Hospital, between years 1999 and 2007.

### Immunohistochemistry (IHC)

IHC was performed using the Bond-Max automated system (Leica Microsystems) according to manufacturer’s instruction. The TMAs were first deparaffinized, followed by heat-induced antigen retrieval solution (pH6.0) for 20 min. Sections were stained with PRDX3 antibody (1:100; Abcam) or MMP-1 antibody (1:100, Proteintech Group). Visualization was achieved using diaminobenzidine (DAB) staining with haematoxylin counterstaining. Negative controls were included by omitting the primary antibody during the procedure. Immunoscoring was done by a researcher and a pathologist blinded to the clinicopathological and survival data. Positive staining of PRDX3 (in the epithelial cells) and MMP-1 (in both the epithelial and stromal cells) was assessed and expression levels defined by the total percentage of positive cells stained. Due to the loss of individual tissue sections during the procedure, a total of 152 cases of IDC and 32 cases of adjacent normal breast counterpart were finally used for the analysis.

### Statistical analysis

For analysis of in vitro experimental data, the GraphPad Prism software (San Diego, CA) was used. At least 3 independent experiments were carried out and data were expressed as mean ± SEM. Unpaired t-test was used for comparison between two groups. For IHC data analysis, the PASW Statistic 18 software for Window (SPSS, Inc.) was used. The difference between adjacent normal and breast tumor tissues were compared using Mann-Whitney non-parametric test. Correlation of PRDX3 expression with MMP1 epithelial and stromal cells expression was determined using Spearman’s correlation test. *p* < 0.05 was considered as statistically significant.

## Results

### PRDX3 is upregulated in breast cancer and knockdown of PRDX3 attenuated cell migration and invasion

**Fig. 1:**
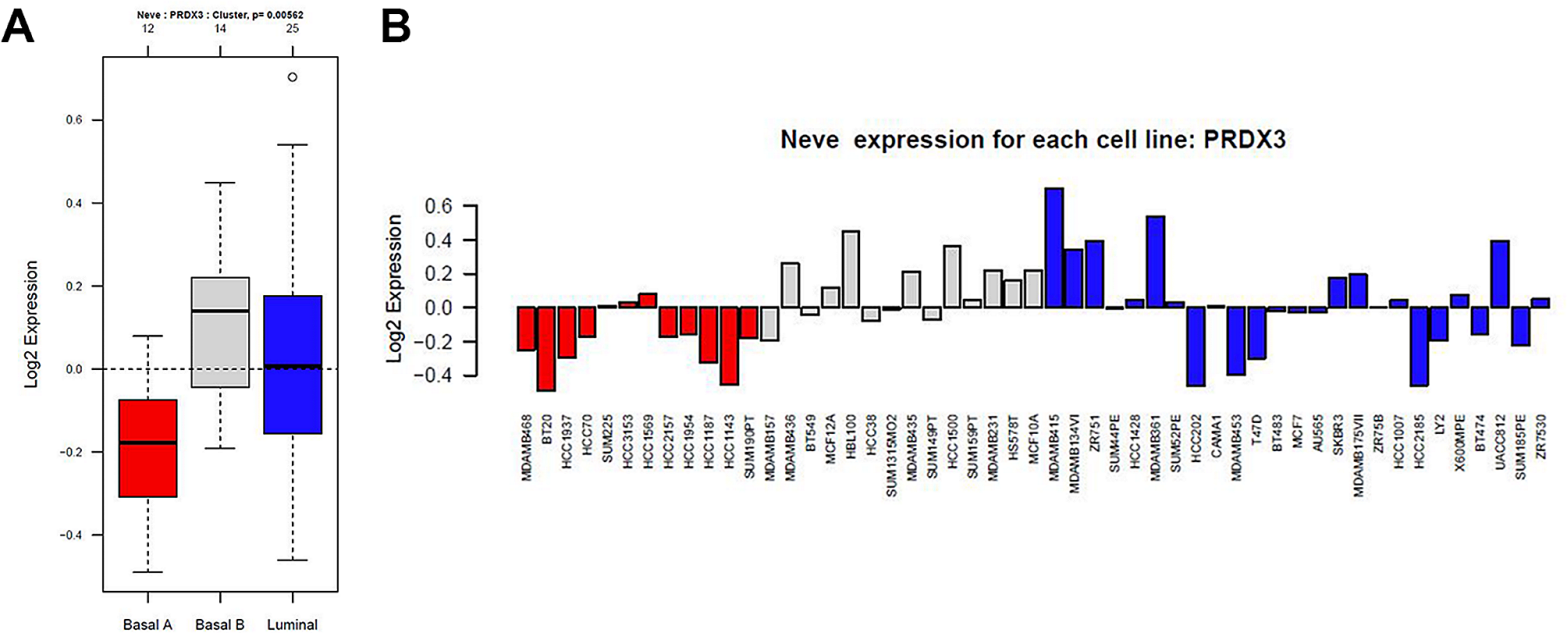
PRDX3 mRNA expression in breast cancer cell lines. (**A**) Box plots showing PRDX3 gene expression in human breast cancer cell lines according to basal A (red), basal B (grey) and luminal (blue) subtypes. (**B**) Gene expression analysis of PRDX3 in 51 breast cancer cell lines

According to the Gene expression-based Outcome for Breast cancer Online (GOBO) analysis [22, 23], the PRDX3 gene is highly expressed in basal B breast cancer cell lines in comparison with the basal A and luminal subtypes (Fig. 1A, B). As basal B breast cancer cell lines are often associated with tumor invasive and aggressive features, two triple negative breast cancer cell lines, MDA-MB-231 and BT-549 (with baseline PRDX3 expression shown in Figure S1) were selected for further investigations.

**Fig. 2:**
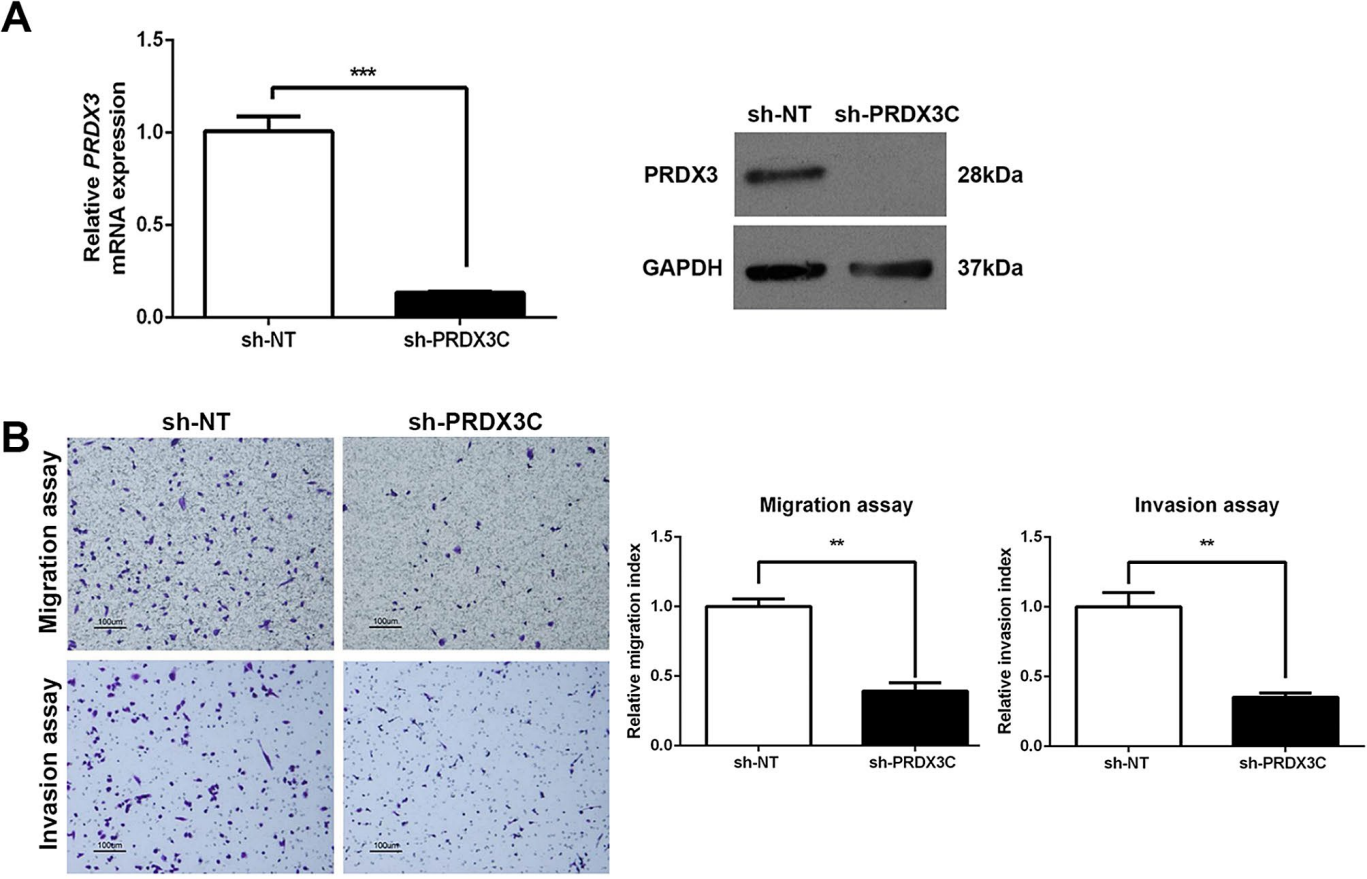
Stable PRDX3 knockdown decreases cell migration and invasion in MDA-MB-231 breast cancer cells. (**A**) Bar chart of PRDX3 mRNA expression by real-time RT-PCR (left panel) and western blot of PRDX3 protein (right panel) indicating successful knockdown of PRDX3 in MDA-MB-231 cell line. (**B**) Representative microscopic field of migrated and invaded cells at 100X magnification (left panel). Knockdown of PRDX3 significantly attenuated breast cancer cell migration and invasion. Experiment data are expressed as mean ± SEM; *p < 0.05, **p < 0.01, ***p < 0.001

**Fig. 3:**
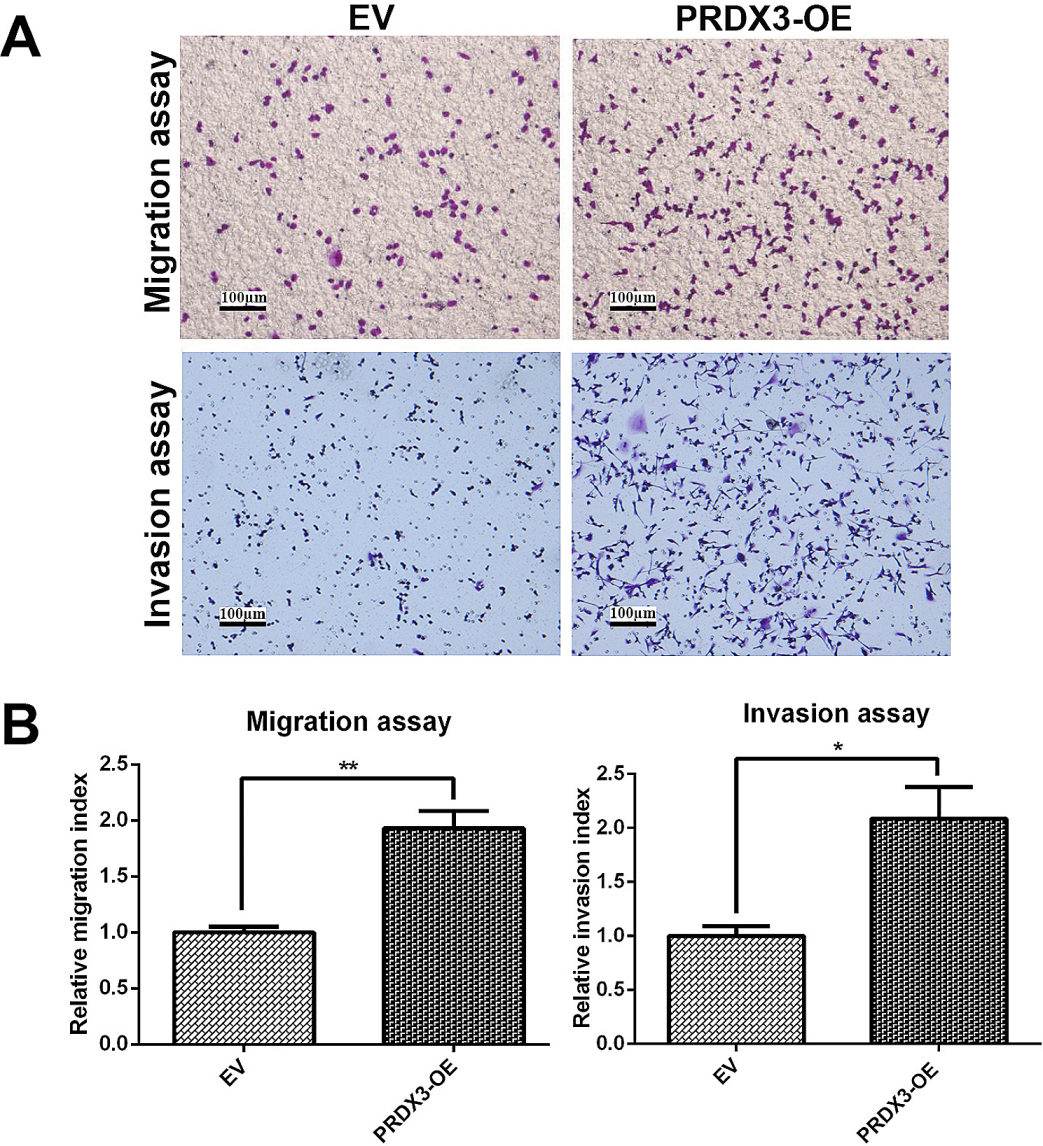
PRDX3 overexpression enhances cell migration and invasion in control (EV) and PRDX3-overexpressing (PRDX3-OE) MDA-MB-231 breast cancer cells. (**A**) Representative microscopic field of migrated and invaded cells at 100X magnification. (**B**) Bar chart showing the quantitative analysis of the migration and invasion assay of the cells. Each bar represents mean ± SEM, *n* = 3. **p < 0.01, ***p < 0.001

Stable shRNA-mediated silencing of the *PRDX3* (Fig. 2A) showed a significant reduction in the cell migratory and invasive potential in MDA-MB-231 breast cancer cells (Fig. 2B). Meanwhile, depletion of PRDX3 expression by transient siRNA (Fig S2A) significantly reduced cell invasion but had an equivocal effect on migration in BT-549 breast cancer cells (Fig S2B). To further confirm the results, a reciprocal experiment was performed. Overexpression of PRDX3, on the other hand, significantly enhanced migration and invasion in MDA-MB-231 breast cancer cells (Fig. 3A, B). Taken together, these results suggest that PRDX3 regulate fundamental processes involved in the metastatic cascade, may therefore play a crucial role in the metastasis of breast cancer.

### PRDX3 regulates total MMP-1 at cellular level and active MMP-1 secretion into the extracellular matrix

**Fig. 4:**
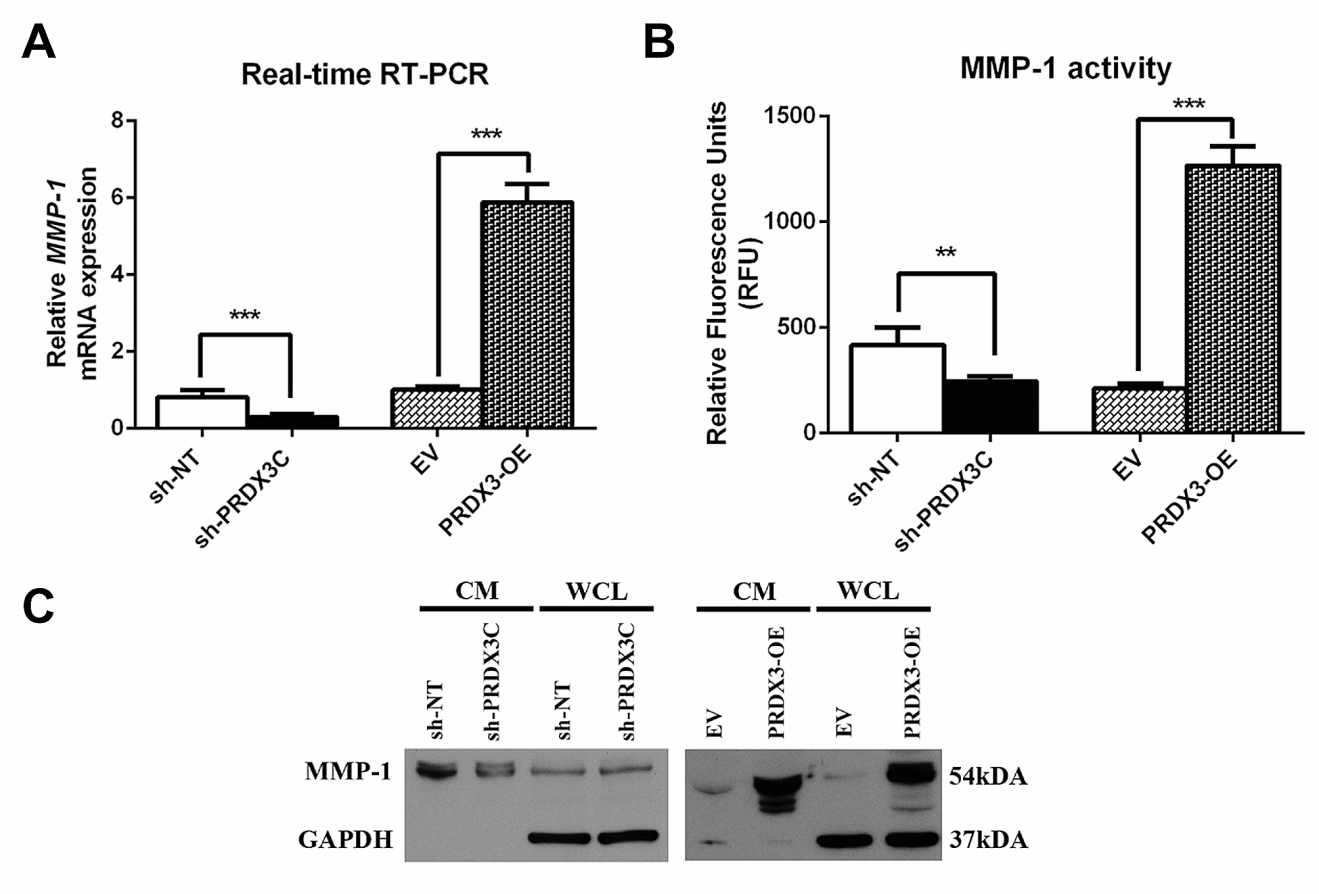
MMP1 plays an important role in PRDX3-mediated breast cancer tumorigenesis. (**A**) Real-time RT-PCR of the mRNA levels of MMP1 in sh-NT, sh-PRDX3C, EV and PRDX3-OE stable transfected cells. (**B**) Enzyme-linked immunosorbent assay (ELISA) of MMP1-secrected activity was evaluated in conditioned medium (CM). (**C**) Western blot analysis of MMP1 protein level in conditioned medium (CM) and whole cell lysates (WCL). GAPDH was used as loading control for WCL. Each bar represents mean ± SEM, *n* = 3. **P < 0.01, ***P < 0.001

Previous studies have reported that expression of MMP-1 is associated with invasive behavior in breast cancer [24, 25]. We therefore examined the expression of MMP-1 using real-time-RT-PCR, western blot, and an enzyme-linked immunosorbent assay (ELISA) and explored the possibility of a relationship between PRDX3 and MMP-1. MMP1 was observed to be significantly suppressed in the shPRDX3C group, while overexpression of PRDX3 enhanced its mRNA expression (Fig. 4A). The conditioned medium was collected, and the activity levels of active MMP-1 secreted into culture medium was analyzed. The results showed a 2.4-fold reduction of active MMP-1 activity in PRDX3 knockdown group, while a 4.8-fold increase in its activity in PRDX3 overexpressed group, as compared to the respective control (Fig. 4B). In addition, the MMP-1 protein in both conditioned medium and whole cell lysate was down-regulated in PRDX3 knockdown MDA-MB-231 cells compared to control cells, but up-regulated in cells overexpressing PRDX3 compared to the respective EV control cells (Fig. 4C).

### PRDX3 regulates MMP-1 via AP-1 transcriptional activity

MMP-1 expression and its activity can be regulated via several mechanisms: primarily at transcriptional level [26, 27], and post-transcriptional level such as activation of zymogen or negative regulation by enzyme inhibitors [28, 29]. Therefore, a cellular signaling pathways reporter assay was applied to identify potential transcription factor(s) activity.

**Fig. 5:**
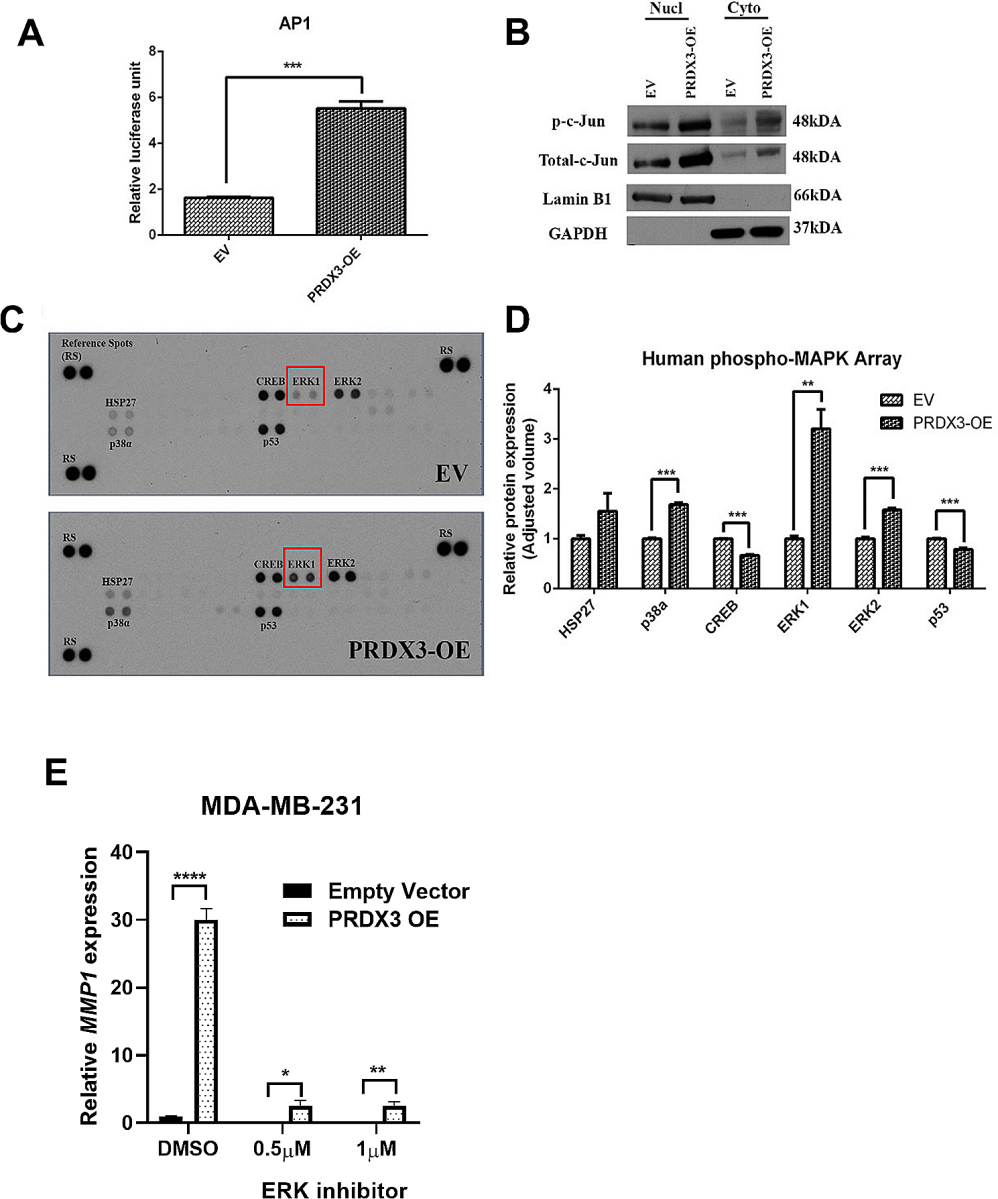
PRDX3 regulates MMP1 via MAPK. (**A**) Multi-pathway reporter array showed the transcriptional activities change of AP-1 in PRDX3-OE group (refer to Supplementary Figure for full investigated pathways). Relative luciferase activity was denoted as the ratio of firefly luciferase to renilla luciferase values. (**B**) Representative western blots showing protein expression of p-c-Jun, total c-Jun. Lamin B1 was used as nuclear loading control while GAPDH was used as cytoplasmic fraction control. (**C**) Human phospho-MAPK proteome profiler array demonstrates PRDX3 induced phosphorylation of MAPK and other serine/threonine kinases. Data is representative of a single experiment out of two experiments done. (**D**) Graph bars depicted the relative protein expression levels of several phosphorylated proteins in PRDX3-OE samples. (**E**) Treatment with SCH772984, an ERK inhibitor, for 24 h inhibited MMP1 expression in PRDX3-overexpressing MDA-MB-231 cells. Each bar represents mean ± SEM, **P < 0.01, ***P < 0.001, ****P < 0.0001

Numerous transcriptional activities, such as Nrf2/Nrf1, NF-kB, HIF1, CBF/NF-Y-YY1, HSF1, AP1 and AhR exhibited significant differential changes in PRDX3 overexpression cells (Fig S3). By using the transcription factor database search (http://www.sabiosciences.com/chipqpcrsearch.php), activator protein-1 (AP-1) binding site was noted to be a common promoter site present in MMP-1. As expected, overexpression of PRDX3 significantly stimulated a more than 3.3-fold induction of AP-1 transcriptional activity (Fig. 5A).

The expression and phosphorylation of c-Jun, one of the major components of AP-1 transcription complex, was also examined by western blot. As shown in Fig. 5B, overexpression of PRDX3 elevated the active phosphorylation of c-Jun. Moreover, activation of c-Jun is also known to contribute to cell invasion and metastasis in various cancers. Furthermore, PRDX3 overexpression also induced phosphorylation of MAPK and other serine/threonine kinases, which are important in cancer metastasis (Fig. 5C, D). Importantly, treatment with SCH772984, an ERK inhibitor, [30] reduced MMP1 expression in PRDX3-overexpressing MDA-MB-231 cells (Fig. 5E). Taken together, through maintaining and promoting the oncogenic AP-1 activation, PRDX3 may contribute to the activation of AP-1 downstream signaling pathways target MMP-1, that is critical for the malignancy of human breast cancer cells.

### PRDX3 is positively associated with MMP-1 expression in breast cancer samples

**Fig. 6:**
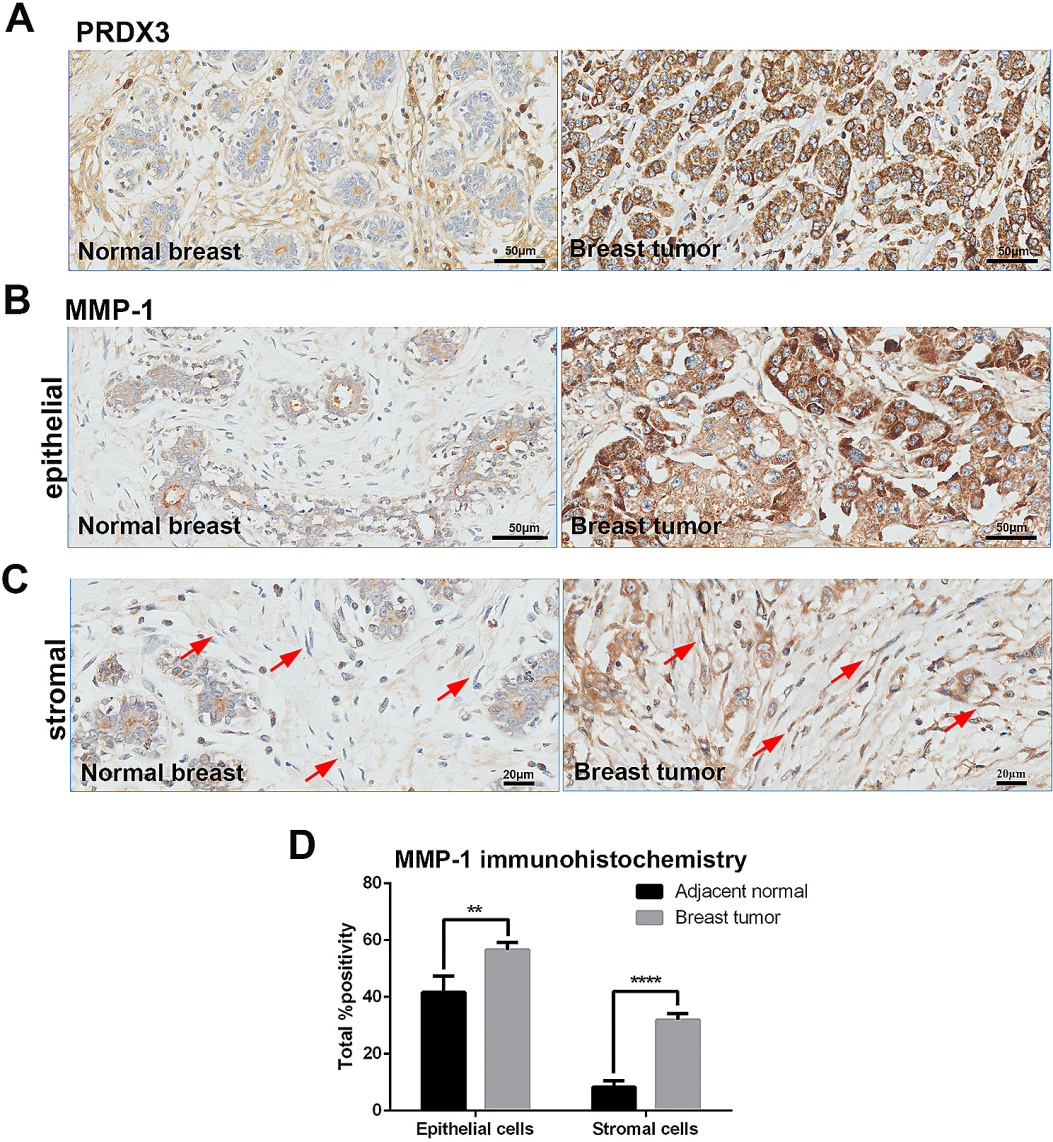
Immunohistochemistry analysis of PRDX3 and MMP-1 in 152 pairs of breast cancer tissues. Representative image of (**A**) PRDX3 and (**B**, **C**) MMP-1 in epithelial and stromal components (indicated by red arrows) respectively. (**D**) Quantitative analysis showed that MMP-1 expression was significantly higher in both epithelial and stromal components of breast cancer tissues

To validate our results in the clinical setting, we analyzed the expression of PRDX3 and MMP-1 in 152 paired breast cancer patient samples. Expression of MMP-1 was significantly higher in breast cancer tissues, compared to adjacent normal tissues, in both epithelial and stromal parts of the tissue (Fig. 6A-D). Notably, PRDX3 expression was positively correlated with MMP-1 expression in both epithelial (Spearman’s rho = 0.2868, *P* < 0.001) and stromal parts (Spearman’s rho 0.2205, *P* < 0.01) of cancer tissues.

## Discussion

Despite significant advances in breast cancer treatment, metastatic breast cancer is still largely incurable [31]. Due to the limited availability of treatment strategies, distant metastasis remains a major challenge in the clinical setting [32]. Cancer metastasis is regulated by diverse molecular pathways and a better understanding of molecular markers may allow for development of novel and efficacious therapeutic regimes. Our findings show that PRDX3 enhances cellular processes associated with breast cancer spread.

Overexpression of PRDX3 has been documented in a number of cancer types due to alteration of the metabolic state in mitochondria [13, 21, 33–37]. It can also protect cancer cells against chemotherapeutic-induced apoptosis [15, 16]. GOBO analysis also showed that the *PRDX3* gene is differentially expressed in breast cancer cell lines, with the highest expression in basal breast cancer cell lines. Knockdown of PRDX3 expression in triple negative basal B MDA-MB-231 and BT-549 breast cancer cells inhibited cell migratory and invasive potential, demonstrating the oncogenic role of PRDX3 in promoting tumour progression. The role of PDX3 in tumor progression has also been documented in several human cancers [17, 18, 20], however, its molecular function is not fully explored. Our current results also showed that PRDX3 can exert its invasive potential, via upregulation of MMP-1 in breast cancer cells.

MMP-1, also known as Collagenase 1, is an enzyme that plays a critical role in tissue remodeling, wound healing, and extracellular matrix degradation [38]. It belongs to a family of zinc-dependent endopeptidases known as matrix metalloproteinases (MMPs) [29]. MMP-1 specifically targets and breaks down various components of the extracellular matrix, primarily collagen type I, which is a major structural protein in connective tissues such as skin, tendons, and bone [38].

Previous studies showed that MMP-1 is overexpressed in several human cancers [25, 39–41], and is associated with invasive behavior and tumor progression [42]. In this study, we observed that PRDX3 contributed to the invasive ability in two breast cancer cell lines, via upregulation of MMP-1, which is due to the activation of ERK/AP1 signaling, as demonstrated by multi-pathway reporter array and western blotting. Although previous studies have also demonstrated that MMP-1 can be upregulated by activation of ERK [43, 44], this is the first report to our knowledge, demonstrating PRDX3 can activate MMP-1 via ERK signaling. In this regard, administration of an ERK specific inhibitor was shown to reduce MMP-1 expression, and possibly metastasis in PRDX3-overexpressing breast cancer. Although the precise mechanism of how PRDX3 activates ERK is not fully understood, a recent study has demonstrated that overexpression of SRXN1, an endogenous antioxidant protein, can upregulate PRDX3, and activate ERK signaling as an anti-oxidative response in cardiomycoytes [45]. It is plausible that PRDX3 could also upregulate MMP-1 via activation of ERK signaling in a similar manner.

However, Liu et al. showed that silencing of PRDX3 inhibits proliferation but promotes invasion in human hepatocellular carcinoma (HCC) cells, via downregulation of TIMP-1 [17]. Recently Yu et al. also demonstrated that Praeruptorin A, a natural product found in Ligusticum lucidum, can downregulate MMP-1, via activation of ERK in human HCC cells [46]. These results suggest that the role of PRDX3 and ERK signaling pathways in modulating MMP-1 expression can be breast cancer cell-type dependent, with more work needed to clarify the dichotomy observed.

There are some limitations in this present study, Firstly, the exact role of PDXD3 in ERK activation is still not fully explored. Proteomic or transcriptomic analyses are required to further understand how PRDX3 contribute to the tumor progression. Secondly, we did not perform any in vivo experiments, which could provide additional insights pertaining to the role of PRDX3 in pre-clinical models of breast cancer progression.

In conclusion, the present work demonstrates that PRDX3 plays an essential role in promoting breast cancer migration and invasion, via ERK-mediated upregulation of MMP-1. To our knowledge, this is the first time that PRDX3 is shown to induce MMP-1 expression in breast cancer. Targeted inhibition of ERK signaling may be able to inhibit metastasis in PRDX3-overexpressed breast cancer- a personalized oncology approach that would advance individualization of breast cancer therapy [47].

### Electronic supplementary material

Below is the link to the electronic supplementary material.

**Supplementary Material 1**: **Figure S1**. PRDX3 expression analysis in MDA-MB-231 and BT-549 breast cancer cell lines. **Figure S2**. Transient siRNA-mediated PRDX3 knockdown decreases BT-549 cell migration and invasion. **Figure S3**. Multi-pathway reporter array showed the transcriptional activities change in PRDX3-overexpressed MDA-MB-231 cell line.

**Supplementary Material 2**: **Table S1.** Dilution of primary and Secondary antibodies used in this study.
