# Supplementary material for: Peroxiredoxin 3 regulates breast cancer progression via ERK-mediated MMP-1 expression
Source: Cancer Cell Int. 2024 Feb 6;24:59. doi: 10.1186/s12935-024-03248-x (PMC10845805; doi:10.1186/s12935-024-03248-x)
Supplement: Supplementary file 1 — Supplementary Material 1: Figure S1. PRDX3 expression analysis in MDA-MB-231 and BT-549 breast cancer cell lines. Figure S2. Transient siRNA-mediated PRDX3 knockdown decreases BT-549 cell migration and invasion. Figure S3. Multi-pathway reporter array showed the transcriptional activities change in PRDX3-overexpressed MDA-MB-231 cell line. [file 12935_2024_3248_MOESM1_ESM.pdf]

Supplementary Figures

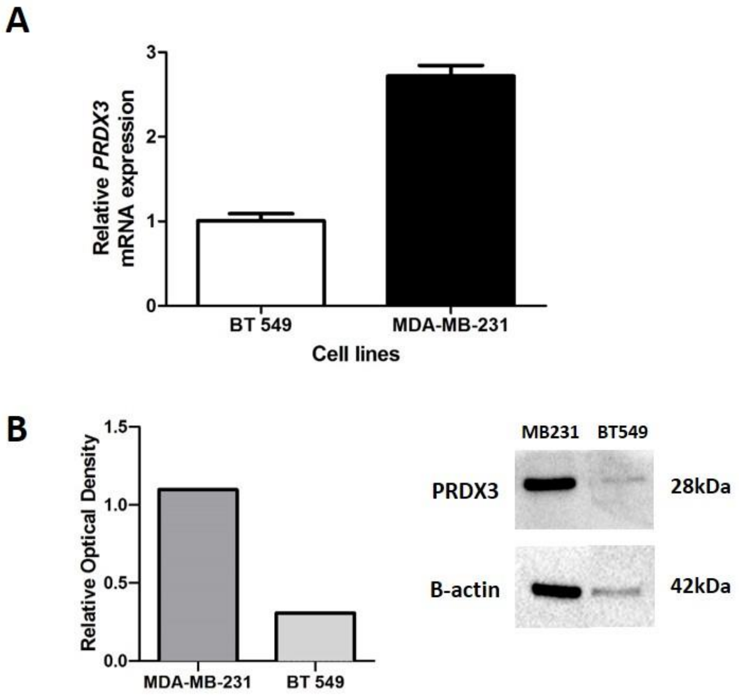

Figure S1. PRDX3 expression analysis in MDA-MB-231 and BT-549 breast cancer cell lines. (A) Relative expression of *PRDX3* gene by normalization to *GAPDH* housekeeping gene using real-time RT-PCR. (B) PRDX3 protein expression in both cell lines were analyzed by immunoblotting with antibodies against PRDX3 and β-actin.

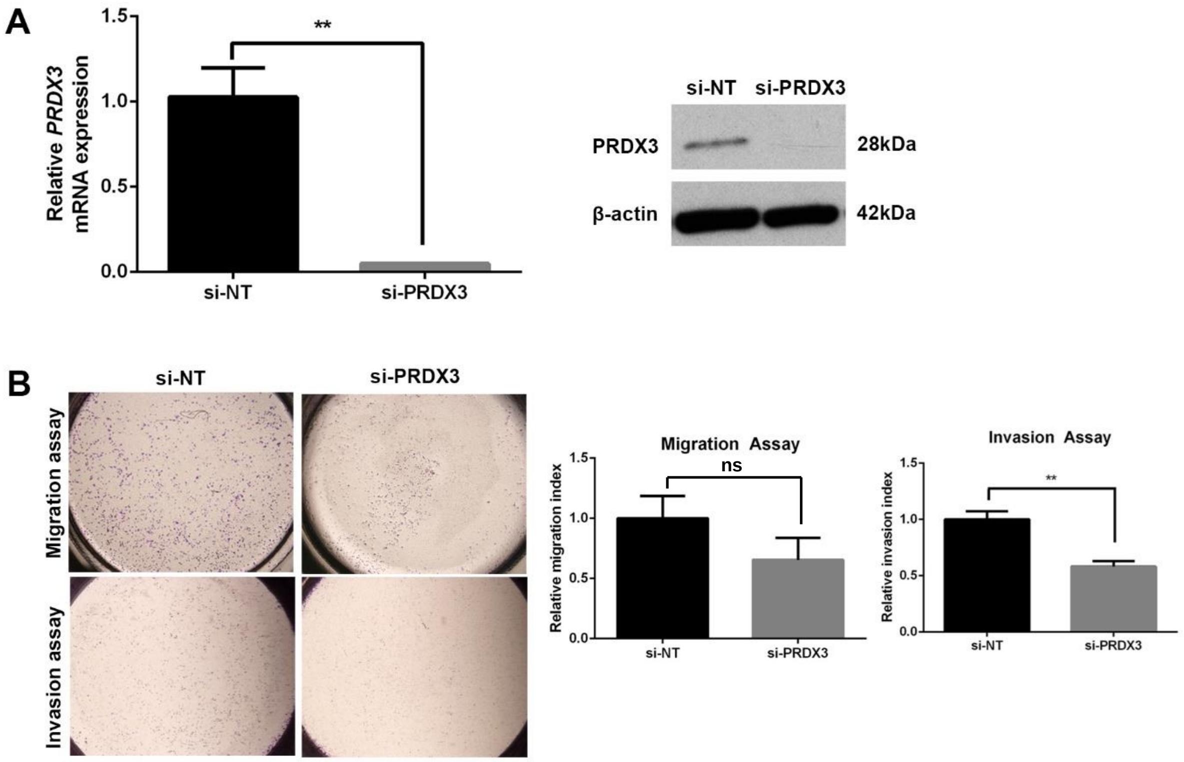

Figure S2. Transient siRNA-mediated PRDX3 knockdown decreases BT-549 cell migration and invasion. (A) Bar chart of *PRDX3* mRNA expression by real-time RT-PCR (left panel) and western blot of PRDX3 protein (right panel) indicating successful knockdown of PRDX3 in BT-549 cell line. (B) The migratory properties of BT-549 was reduced after PRDX3 knockdown, despite no statistical significance was observed. Knockdown of PRDX3 attenuated the invasive capability of BT-549 breast cancer cell line. Both assays were performed as described in Materials and Methods. Data is expressed as mean ± SEM; **P < 0.01.

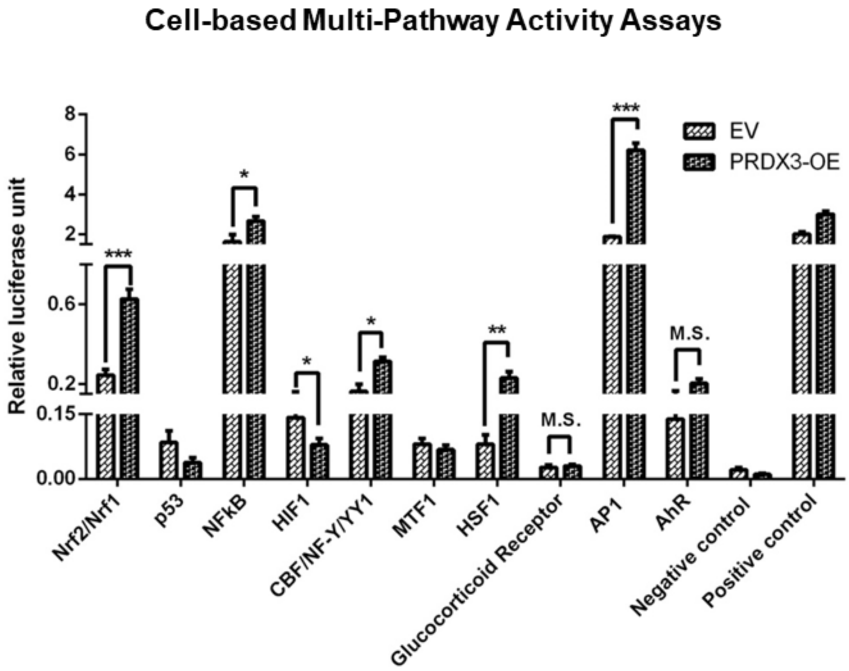

Figure S3: Multi-pathway reporter array showed the transcriptional activities change in PRDX3-overexpressed MDA-MB-231 cell line. Relative luciferase activity was denoted as the ratio of firefly luciferase to renilla luciferase values. Each bar represents mean ± SEM. M.S. represents marginal significant *P*-value, **P*<0.05, ***P*<0.01, ****P*<0.001. Nrf, Nuclear respiratory factor; NF-kB, Nuclear factor-κB; HIF1, Hypoxia-inducible factor 1; CBF, CCAAT-binding factor; NF-Y, Nuclear factor Y; YY1, Yin Yang 1; MTF1, Metal regulatory transcription factor 1; HSF1, Heat shock factor 1; AP1, Activator protein 1; AhR, Aryl hydrocarbon receptor.
