# Supplementary material for: Peroxiredoxin 3 regulates breast cancer progression via ERK-mediated MMP-1 expression
Source: Cancer Cell Int. 2024 Feb 6;24:59. doi: 10.1186/s12935-024-03248-x (PMC10845805; doi:10.1186/s12935-024-03248-x)
Supplement: Supplementary file 2 — Supplementary Material 2: Table S1. Dilution of primary and Secondary antibodies used in this study. [file 12935_2024_3248_MOESM2_ESM.pdf]

Supplementary Table S1: Dilution of primary and Secondary antibodies used in this study.

**Protein**	**Primary Antibody Dilution**	**Type**	**Supplier**	**Secondary Antibody Dilution** (Sigma-Aldrich)
β-actin	1:6000	Mouse monoclonal	Sigma-Aldrich	1:10,000
PRDX3	1:1000	Rabbit polyclonal	Abcam	1:5000
Phospho-c-Jun	1:1000	Rabbit Polyclonal	Cell Signalling Technology	1:2000
Total c-Jun	1:1000	Rabbit Polyclonal	Cell Signalling Technology	1:2000
GAPDH	1:2000	Mouse monoclonal	Santa Cruz Biotechnology	1:5000
MMP-1	1:2000	Rabbit Polyclonal	Proteintech Group	1:10,000
Lamin B1	1:2000	Mouse monoclonal	Proteintech Group	1: 5000
